# Time to progression to castration-resistant prostate cancer after commencing combined androgen blockade for advanced hormone-sensitive prostate cancer

**DOI:** 10.18632/oncotarget.26426

**Published:** 2018-12-11

**Authors:** Satoshi Tamada, Taro Iguchi, Minoru Kato, Jumpei Asakawa, Kazuaki Kita, Sayaka Yasuda, Takeshi Yamasaki, Yudai Matsuoka, Kazuyuki Yamaguchi, Kentaro Matsumura, Ishun Go, Tetsuji Ohmachi, Tatsuya Nakatani

**Affiliations:** ^1^ Department of Urology, Osaka City University Graduate School of Medicine, Abeno-ku, Osaka 545–8585, Japan; ^2^ Department of Urology, Bell Land General Hospital, Naka-ku, Sakai City, Osaka 599–8247, Japan

**Keywords:** castration-resistant prostate cancer, combined androgen blockade, hormone-sensitive prostate cancer, progression, survival

## Abstract

**Purpose:**

The aim of our retrospective study was to determine the time to progression to castration-resistant prostate cancer (CRPC) in prostate cancer patients who undergo combined androgen blockade (CAB), as well as their prognoses.

**Materials and Methods:**

We examined the overall survival (OS) and disease-specific survival rates, as well as the time to CRPC development, in 387 patients who were treated with CAB for prostate cancer. The disease-specific survival rate and time to CRPC were stratified by prostate-specific antigen (PSA) levels, Gleason score (GS), and presence of metastasis at diagnosis. We designated high-risk patients as those satisfying at least two of the following three criteria: extent of disease of bone metastasis grade ≥2, presence of metastasis at diagnosis, and a GS ≥8.

**Results:**

The 10- and 15-year OS rates were 74.0% and 50.4%, respectively, while the corresponding disease-specific survival rates were both 86.8%. Metastasis at diagnosis was an independent prognostic factor for disease-specific survival. The median time to CRPC development was 140.7 months. A PSA level ≥20 ng/mL, a GS ≥8, and the presence of metastasis at diagnosis were independent predictors of a shorter time to CRPC development. The 10-year disease-specific survival rate in the high-risk group was significantly lower than that in the low-risk group (approximately 74% vs. 98%), and the time to CRPC development was significantly shorter (median: 20.5 months vs. not reached).

**Conclusions:**

The time to CRPC development was shorter in high-risk prostate cancer patients with metastases. Such patients require alternative novel treatment modalities.

## INTRODUCTION

Since Huggins first reported that surgical castration is an effective treatment for advanced prostate cancer [1], hormonal therapy has become an established intervention for previously untreated patients with this disease. In 1982, Labrie *et al.* indicated the need for anti-androgen drug administration concomitant with surgical or medical castration [2]. Since then, androgen deprivation therapy (ADT) and combined androgen blockade (CAB) have been commonly used for the treatment of locally advanced or metastatic prostate cancer. CAB was not found to be superior to ADT monotherapy in Western countries [3]; however, controlled trials in Japan found that CAB led to significantly longer overall survival (OS) than ADT monotherapy (hazard ratio [HR]: 0.78) in patients with stages C and D1 disease, although not in those with stage D2 disease [4, 5]. As a result, CAB is frequently administered to Japanese prostate cancer patients [6]. In Western countries, the Systemic Therapy in Advancing or Metastatic Prostate Cancer: Evaluation of Drug Efficacy (STAMPEDE) trial found that the failure-free survival in patients receiving ADT monotherapy was only 11 months [7]. Moreover, OS following ADT monotherapy was 24 months among patients with prostate cancer who had bone metastasis at initial diagnosis and 16 months among those who had visceral metastasis; the median OS of those who had both types of metastasis was 14 months [8]. Since these data indicate that ADT is not sufficiently effective for patients with metastatic prostate cancer, new treatment methods have been explored. Some studies showed that docetaxel or abiraterone in combination with initial hormonal therapy was more effective for high-risk prostate cancer patients than ADT monotherapy [9–12]. In these studies, however, ADT monotherapy was used in all the control groups, whereas in Japan, CAB is often used as the initial hormonal therapy. Therefore, it remains unclear whether ADT combined with docetaxel or abiraterone is beneficial for Japanese patients.

Few studies have investigated the time to progression to castration-resistant prostate cancer (CRPC) following the initiation of hormonal therapy. In the ‘Chemo-Hormonal Therapy versus Androgen Ablation Randomized Trial for Extensive Disease in Prostate Cancer’ (CHAARTED) trial [9], Sweeny *et al.* found that the median time to biochemical, symptomatic, or radiographic progression was 11.7 months in patients receiving ADT monotherapy. Hinotsu *et al.* reported clinically acceptable progression-free survival and OS in prostate cancer patients treated with ADT [13]. However, they did not investigate the time to transition from hormone-naive prostate cancer to CRPC.

Therefore, in our current study, we focused on prostate cancer patients who initially underwent hormonal therapy (particularly CAB) and examined their prognoses and time to progression to CRPC. Furthermore, we evaluated these same endpoints specifically in high-risk prostate cancer patients to determine the effectiveness of CAB.

## RESULTS

Table 1 summarizes the patients’ characteristics. The 10-year and 15-year OS rates were 74.0% and 50.4%, respectively (median: not reached). Forty-eight patients died of any cause during the follow-up period. Furthermore, the 10-year and the 15-year disease-specific survival rates were both 86.8% (median: not reached). Twenty-one patients died during the follow-up period due to prostate cancer.

**Table 1:** Patients characteristics (*N* = 387)

Age in years, median (range)	77	(49–95)
Observation period in months, median (range)	33.3	(0.4–230.1)
PSA at initial diagnosis in ng/mL, median (range)	41	(2.2–9675)
Gleason score at initial diagnosis (%)		
≤6	32	(8.2)
7	71	(18.3)
≥8	265	(68.5)
unknown	19	(5.0)
T stage (%)		
1	31	(8.0)
2	164	(42.4)
3	122	(31.5)
4	61	(15.8)
unknown	9	(2.3)
Presence of metastatic lesion(s) (%)		
Yes	159	(41.1)
No	228	(58.9)
Metastatic site		
Bone	151	
Distal lymph node	23	
Lung	20	
Others	3	
Extent of disease of bone metastasis		
1	74	
(<3 bone lesions)	(52)	
(≥3 bone lesions)	(22)	
2	54	
3	19	
4	5	

Abbreviations: PSA, prostate-specific antigen.

**Figure 1:**
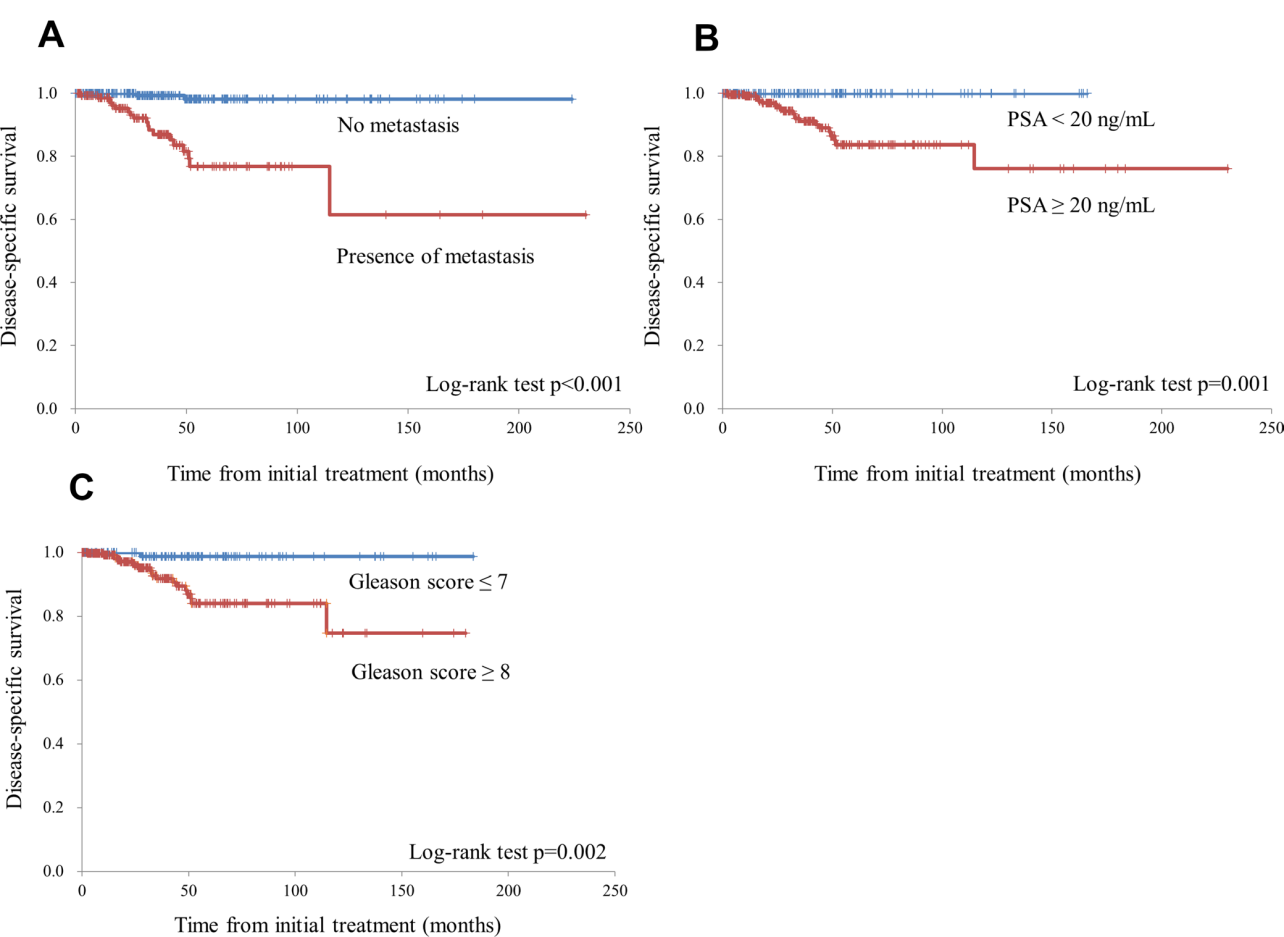
The disease-specific survival rate was stratified by the presence of metastatic foci (**A)**, prostate-specific antigen (PSA) levels at diagnosis (<20 ng/mL vs. ≥20 ng/mL) (**B**), and the Gleason score (≤7 vs. ≥8) (**C**).

Disease-specific survival was stratified by the prostate-specific antigen (PSA) level at diagnosis (<20 vs. ≥20 ng/mL), Gleason score (GS) (≤7 vs. ≥8), and the presence of metastatic foci (Figure 1). Disease-specific survival was significantly shorter among patients with initial PSA ≥20 ng/mL, those with a GS ≥8, and those with metastasis at initial diagnosis. Multivariate analysis revealed that the presence of metastasis at initial diagnosis was an independent negative prognostic factor (Table 2).

**Table 2:** Cox proportional stepwise multivariate analysis for the association between the variables and disease-specific survival

						Unadjusted		Adjusted	
Comparison			Ten-year disease-specific survival in months			HR (95% CI)	*p*-value	HR (95% CI)	*p*-value
Presence of metastasis at initial diagnosis	vs	No metastasis	61.5%	vs	98.2%	17.7 (4.1–76.2)	<0.001	13.4 (3.0–59.1)	<0.001
PSA ≥20 ng/mL	vs	PSA <20 ng/mL	76.1%	vs	100.0%	NA		NA	NA
Gleason score ≥8	vs	Gleason score ≤7	74.7%	vs	98.7%	12.2 (1.6–90.9)	0.015	7.2 (0.9–56.2)	0.059

Abbreviations: HR, hazard ratio; CI, confidence interval; NA, not applicable; PSA, prostate-specific antigen.

**Figure 2:**
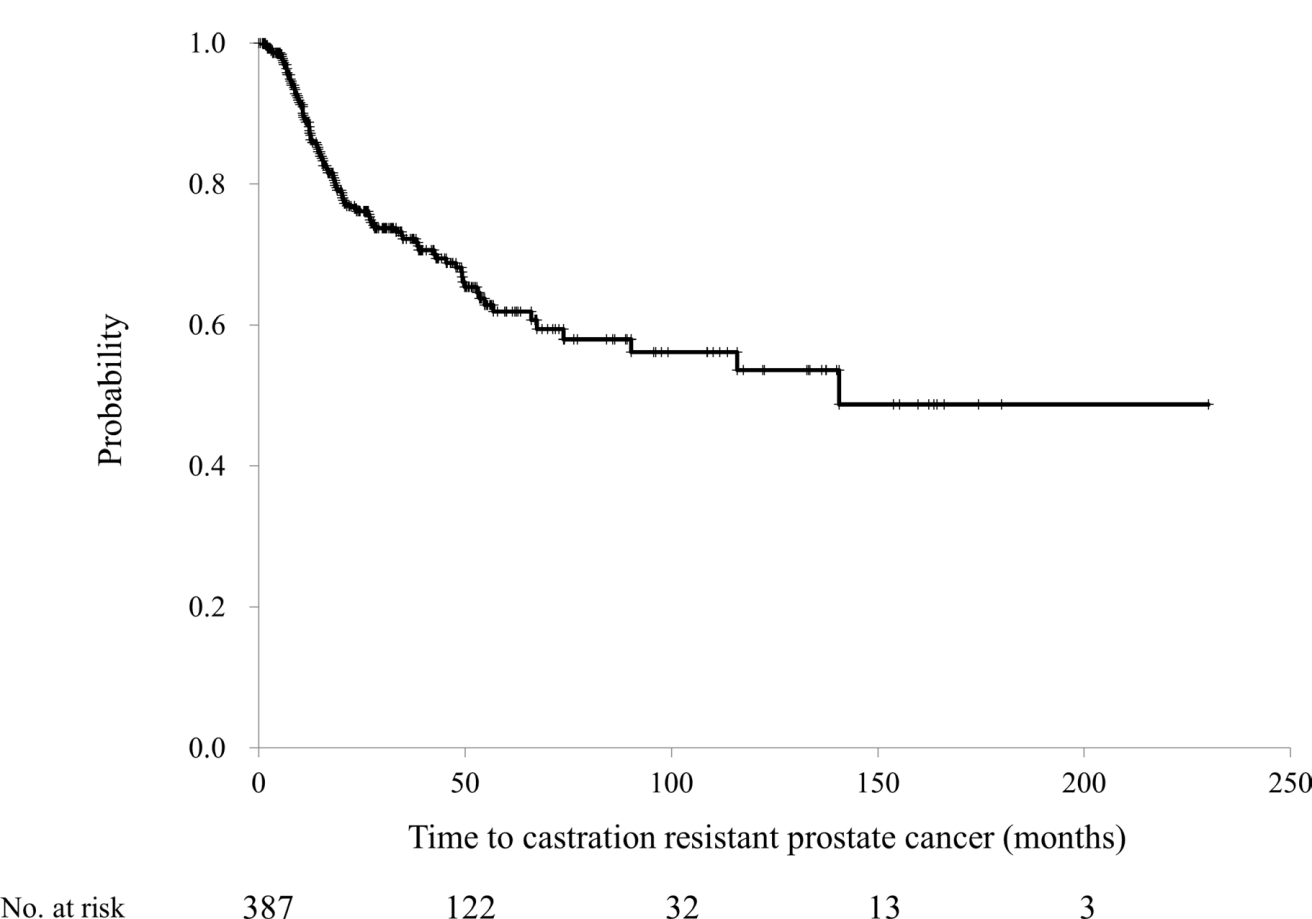
The time to developing castration-resistant prostate cancer among the 387 patients with prostate cancer treated by combined androgen blockade

**Figure 3:**
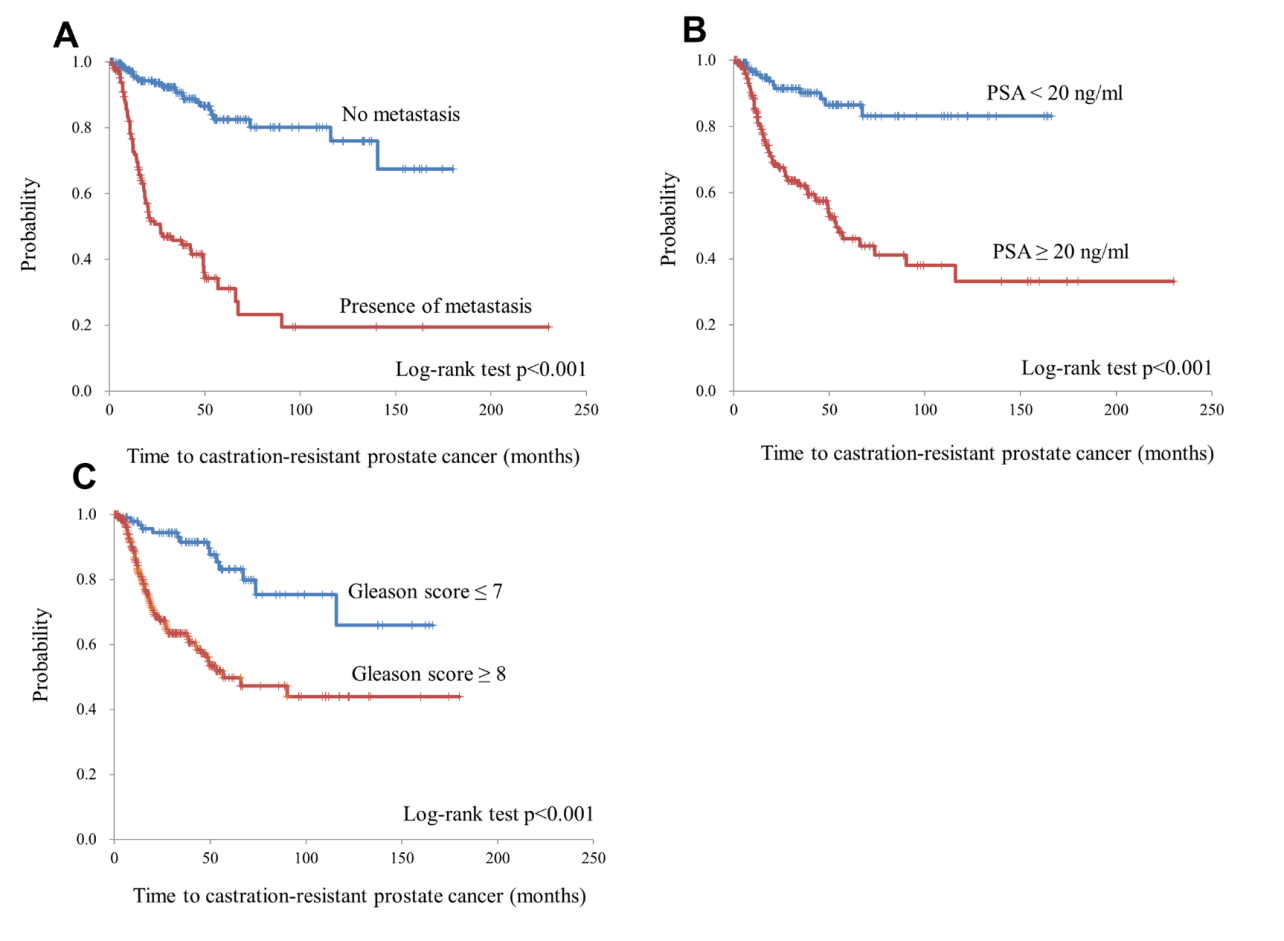
The time to developing castration-resistant prostate cancer was stratified by the presence of metastatic foci (**A**), prostate-specific antigen (PSA) levels at diagnosis (<20 ng/mL vs. ≥20 ng/mL) (**B**), and Gleason score (≤7 vs. ≥8) (**C**).

A total of 105 of the 387 patients progressed to CRPC; the median time to CRPC development was 140.7 months (Figure 2). The time to CRPC development was stratified by PSA levels, GS, and the presence of metastatic foci as described above. The time to CRPC was significantly shorter in patients with PSA ≥20 ng/mL, those with a GS ≥8, and those with metastasis at initial diagnosis (Figure 3); all these variables were found to be independent prognostic factors on multivariate analysis (Table 3).

**Table 3:** Cox proportional stepwise multivariate analysis for the association between the variables and time to CRPC

						Unadjusted		Adjusted	
Comparison			Time to CRPC in months (median)			HR (95% CI)	*p*-value	HR (95% CI)	*p*-value
Presence of metastasis at initial diagnosis	vs	No metastasis	26.6	vs	not reached	7.00 (4.47–10.97)	<0.001	4.79 (2.93–7.83)	<0.001
PSA ≥20 ng/mL	vs	PSA <20 ng/mL	53.5	vs	not reached	4.93 (2.75–8.84)	<0.001	2.48 (1.29–4.73)	0.006
Gleason score ≥8	vs	Gleason score ≤7	56.8	vs	not reached	3.88 (2.19–6.85)	<0.001	2.17 (1.20–3.91)	0.010

Abbreviations: CRPC, castration resistant prostate cancer; HR, hazard ratio; CI, confidence interval; NA, not applicable; PSA, prostate-specific antigen.

**Figure 4:**
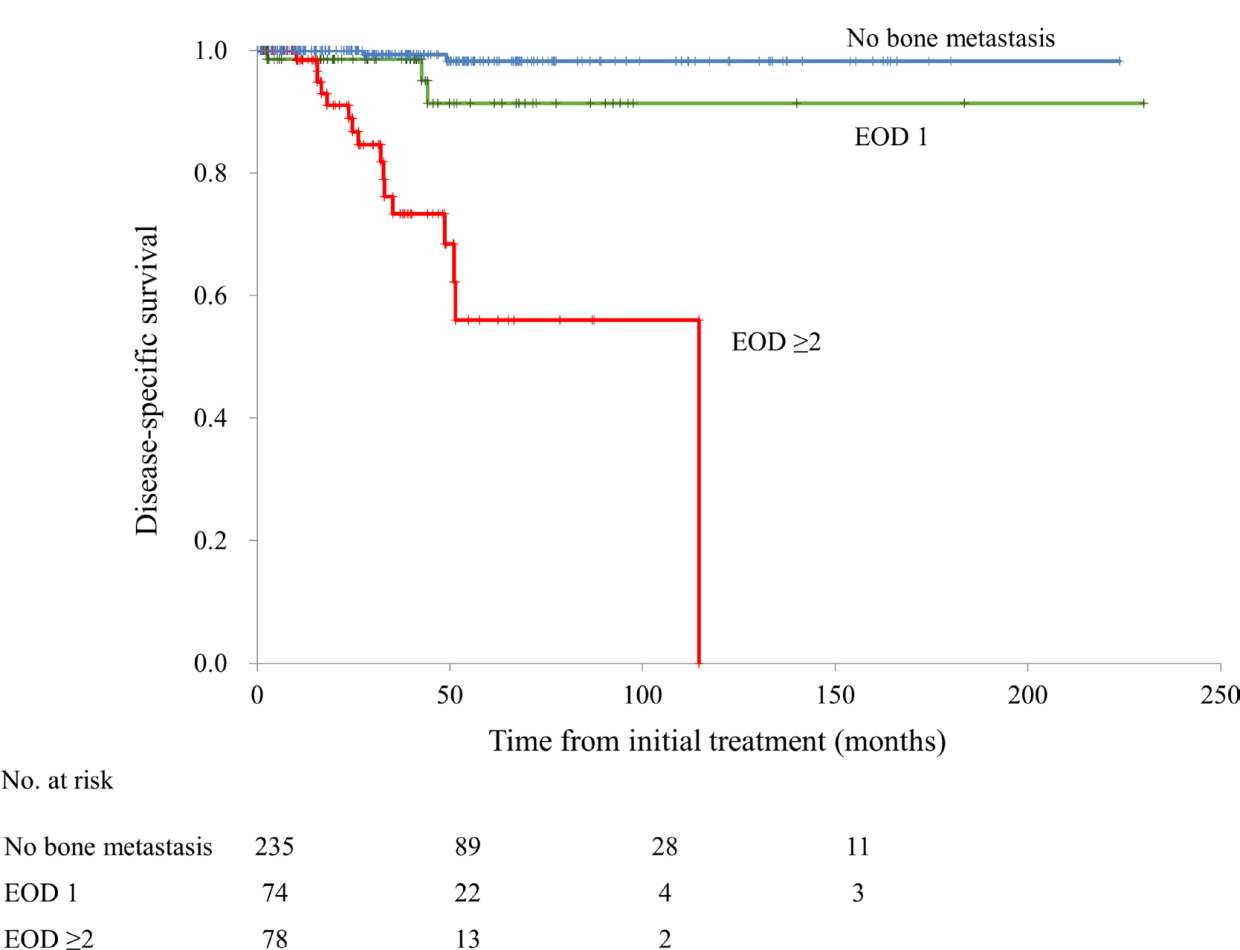
The disease-specific survival rate classified by the extent of disease (EOD) grade (0, 1, or ≥2)

The extent of disease (EOD) of bone metastasis has previously been shown to be closely correlated with OS [14]. Therefore, we analyzed the disease-specific survival rate and time to CRPC development in patients stratified according to EOD grade (0, 1, or ≥2). The Kaplan–Meier curves generated for patients with no bone metastasis and those with EOD grade 1 were similar (Figure 4). Next, we stratified the patients according to EOD grade ≤1 vs. ≥2, and found that disease-specific survival was significantly shorter in patients of the latter group (EOD grades ≤1: median not reached; EOD grades ≥2: median 114.6 months, HR 20.4; 95% confidence interval [CI]: 7.3–56.9; *p* < 0.001). The same was true for patients with CRPC (EOD grades ≤1: median not reached; EOD grades ≥2: median 15.0 months, HR 6.4; 95% CI: 4.3–9.6; *p* < 0.001).

**Figure 5:**
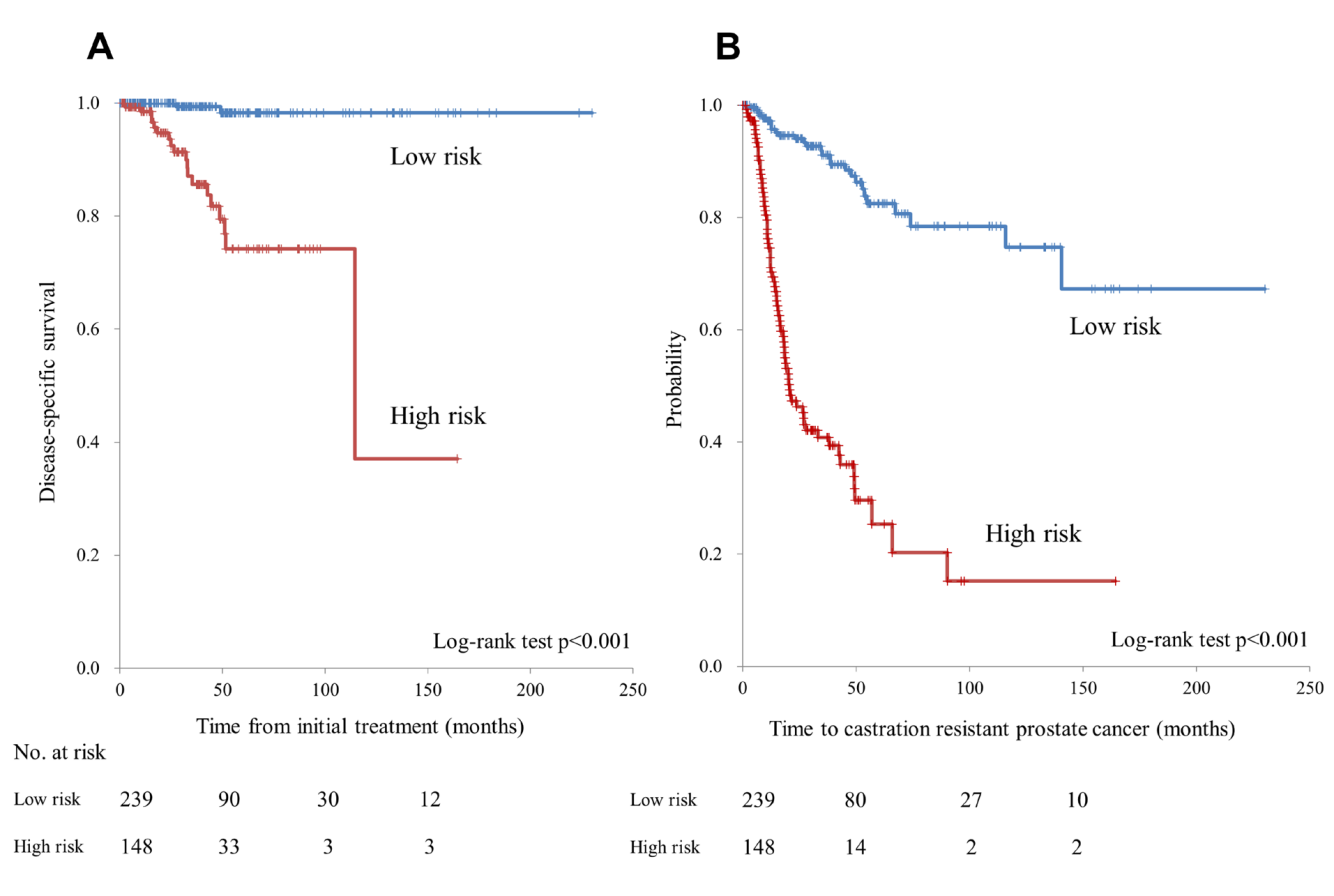
(**A**) Disease-specific survival in the low- and high-risk patient groups. (**B**) Time to progression to castration-resistant prostate cancer in the low- and high-risk patient groups.

In terms of patients with high-risk disease, 72 patients (48.6%) had EOD grade ≥2, metastasis at initial diagnosis, and a GS ≥8; 6 patients (4.1%) had EOD grade ≥2 and metastasis at initial diagnosis; and 70 patients (47.3%) had metastasis at diagnosis and a GS ≥8. Disease-specific survival among patients in this high-risk group was significantly shorter than those in the low-risk group (median: 114.6 months vs. not reached; HR 23.3, 95% CI: 5.3–102.5; *p* < 0.001) (Figure 5A). Similarly, the time to CRPC development was significantly shorter in the high-risk group than in the low-risk group (median: 20.5 months vs. not reached; HR 8.2, 95% CI: 5.3–12.8; *p* < 0.001) (Figure 5B).

Eighty-nine patients (84.8%) proceeded to the next treatment step after being diagnosed with CRPC; they were administered flutamide (*n* = 60), enzalutamide (*n* = 48), abiraterone (*n* = 14), docetaxel (*n* = 17), cabazitaxel (*n* = 8), steroids (*n* = 22), or other drugs (*n* = 5).

## DISCUSSION

To our knowledge, ours is the first study to determine the time to CRPC development in patients who underwent CAB as the initial hormonal therapy for prostate cancer. Overall, the effectiveness of CAB was long-lasting (140 months), but varied markedly according to various risk factors.

Serum PSA level ≥20 ng/mL, a GS ≥8, and presence of metastasis at initial diagnosis were all independent prognostic factors for faster progression to CRPC. In particular, patients with metastasis at initial diagnosis developed castration-resistant disease after a median of 26.6 months, which indicated that the efficacy period of CAB was very short in this population. Since most metastatic foci at initial diagnosis were found in the bones, we also investigated the relationship between the volume of bone metastasis and time to CRPC development. Generally, the volume of bone metastatic foci is expressed on a 5-/point EOD scale [14]. Accordingly, a significant difference in the time to CRPC development was observed between patients with EOD grade 1 and those with EOD grades ≥2. Among patients with bone metastasis, those who have a huge volume of bone metastatic foci may not respond to CAB treatment and may therefore require alternative therapies. Recent studies found that administering docetaxel or abiraterone to early stage hormone-sensitive patients with metastatic prostate cancer prolonged OS [9, 11, 12]. These clinical trials were characteristic in that they all focused on metastatic or high-risk patients, although their definitions of “high-risk” were not consistent. Before our analysis, therefore, we defined “high-risk” patients as those who satisfied at least two of the following three criteria, which were independent risk factors determined using multivariable analysis: the presence of metastasis (visceral and/or bone metastasis) at initial diagnosis, a GS ≥8, and bone metastasis EOD grade ≥2. Serum PSA level was excluded from the analysis because none of the patients with PSA <20 ng/mL died of cancer. We found that both disease-specific survival and time to CRPC development were significantly shorter in the high-risk group than in the low-risk group. In particular, the median time to CRPC development in the high-risk group was a remarkably short 20.5 months. Although comparisons are difficult, these data may indicate that the time to CRPC development following CAB treatment may be shorter than that observed in abiraterone-treated patients in the STAMPEDE and LATITUDE trials [12, 15]. The median age of this study population was 77 years, and the median age of the high-risk group was 75 years. This is an older group than the LATTITUDE trial population. Nevertheless, time to CRPC development was short in the high-risk group; therefore, age may not affect time to CRPC development. Another study examined whether the time to CRPC development could be prolonged by using zoledronic acid in combination with CAB for prostate cancer patients who had bone metastasis at initial diagnosis, but found that the combinational effect was very weak [16]. Taken together, high-risk patients as defined by our criteria may require alternatives to CAB.

The time to CRPC development in the entire high-risk group (20.5 months) was longer than that in patients specifically with EOD grade ≥2 (15.0 months). This is because patients who had both metastasis at initial diagnosis and a GS ≥8 accounted for approximately half of the high-risk group. In other words, the difference can be attributable to the fact that approximately half of the high-risk group comprised patients who had smaller volumes of bone metastatic foci (EOD grade ≤1).

We found that metastasis at initial diagnosis was an independent risk factor of disease-specific survival. Moreover, patients with a GS ≥8 had poorer prognoses than those with a GS ≤7. These findings were similar to those in a controlled trial conducted in Japan to compare CAB and castration monotherapy [5], and supports the possibility that CAB may not be as effective for patients with metastases discovered at initial diagnosis.

Analyses such as ours usually investigate OS; however, we used disease-specific survival because our patients were of older age, with a median age of 77 years, and the death rate from other causes was high (56%). Furthermore, due to the relatively high survival rates, these results should be interpreted with caution. In fact, the median OS was long even in the high-risk patient group (114 months). However, this may partly be because a relatively large number of high-risk patients were lost to follow-up. It should also be noted that the data from high-risk patients were immature because disease-specific death occurred less often. However, since it has been reported that a longer time to CRPC development may correlate with improved OS [17], a longer follow-up period is required.

In summary, the time to CRPC development in patients who underwent CAB as initial therapy and who had metastasis at initial diagnosis was short, as it was for high-risk patients as defined by our criteria. We conclude that CAB treatment is not sufficiently effective for such patients. While the effectiveness of new hormonal drugs to treat CRPC is becoming increasingly evident [18, 19], it remains unknown whether using these drugs for untreated metastatic prostate cancer at an early stage can prolong OS compared to their use at a later stage. Thus, a future prospective study is warranted in this regard.

## PATIENTS AND METHODS

A total of 387 patients who were pathologically diagnosed with adenocarcinoma of the prostate at the Osaka City University Hospital and the Bell Land General Hospital between May 2007 and December 2017 (a 10-year period), were judged not to be candidates for local treatment, and consequently underwent combination therapy as first-line treatment were enrolled in our study. These therapies included gonadotropin-releasing hormone agonists (3.75 mg leuprolide acetate or 3.6 mg goserelin acetate every 4 weeks or 11.25 mg leuprolide acetate or 10.8 mg goserelin acetate every 12 weeks) or gonadotropin-releasing hormone antagonists (initial dose of 240 mg and maintenance dose of 80 mg of degarelix every 4 weeks) as well as anti-androgen drugs (80 mg of bicalutamide orally once a day or 125 mg of flutamide orally 3 times a day) (CAB). Degarelix was discontinued due to adverse events, and a gonadotropin-releasing hormone agonist (leuprorelin acetate or goserelin acetate) was used for maintenance therapy [20]. We examined OS, disease-specific survival, and time to CRPC development in these patients. Disease-specific survival and time to CRPC were stratified by PSA levels at diagnosis (<20 ng/mL vs. ≥20 ng/mL), GS (≤7 or ≥8), and the presence of metastatic foci before performing comparative analyses.

Furthermore, we focused on “high-risk” prostate cancer patients, who were defined as those satisfying two of the following three criteria: EOD of bone metastasis grade ≥2; presence of metastasis at diagnosis (visceral and/or bone metastasis); and a GS ≥8. Patients not meeting at least two of these criteria were considered “low-risk.” On the basis of the number or extent of metastases, patients were divided into five EOD grades based on bone scintigraphy scans as follows: 0, normal or abnormal due to benign bone disease; 1, number of bony metastases less than 6, each of which is less than 50% the size of a vertebral body (one lesion about the size of a vertebral body would be counted as two lesions); 2, number of bone metastases between 6 and 20, size of lesions as described above; 3, number of metastases more than 20 but less than a “super scan”; and 4, “super scan” or its equivalent, i.e., metastasis to more than 75% of the ribs, vertebrae, and pelvic bones [14].

Clinical and histological staging were based on the National Comprehensive Cancer Network Clinical Practice Guidelines in Oncology (version 3, 2016). The definition of CRPC was based on meeting the following European Association of Urology Guidelines criteria: serum testosterone level <50 ng/dL plus 1) successive increases in PSA level during three consecutive measurements obtained ≥1 week apart, an increase of ≥25% on two PSA readings, and a PSA level ≥2.0 ng/mL; or 2) the exacerbation of an existing lesion or development of a new lesion on imaging. OS, disease-specific survival, and the time to CRPC development were estimated using the Kaplan–Meier method, with differences determined using the log-rank test. Cox proportional stepwise multivariate analysis was used to evaluate associations of PSA, GS, and presence of metastatic lesions at initial diagnosis with cause-specific survival and time to CRPC development. A two-sided *p*-value of < 0.05 was considered statistically significant. All statistical analyses were performed using Microsoft Excel^®^ (Microsoft, Redmond, WA, USA). Permission to access the database for a review of the medical records of the patients was obtained from the local research ethics committee at Osaka City University (approval number 4011).

## Abbreviations

ADT: androgen deprivation therapy
CAB: combined androgen blockade
CRPC: castration-resistant prostate cancer
EOD: extent of disease
GS: Gleason score
HR: hazard ratio
OS: overall survival
PSA: prostate-specific antigen
